# MPIDNN-GPPI: multi-protein language model with an improved deep neural network for generalized protein‒protein interaction prediction

**DOI:** 10.1186/s12864-025-12228-y

**Published:** 2025-11-19

**Authors:** Yane Li, Chengfeng Wang, Haibo Gu, Zhentao Long, Ming Fan, Lihua Li

**Affiliations:** 1 College of Mathematics and Computer Science, Zhejiang A&F University, Hangzhou, 311300 China; 2 School of Automation, Hangzhou Dianzi University, Hangzhou, 310018 China

**Keywords:** PPI prediction, Protein language model, Deep neural network, Cross-species

## Abstract

Predicting protein‒protein interactions (PPIs) plays a crucial role in understanding biological processes. Although biological experimental methods can identify PPIs, they are costly, time-consuming, labor-intensive, and often lack stability. In contrast, computational approaches for PPI prediction, particularly deep learning methods, can efficiently learn representations from protein sequences. However, the generalizability, robustness, and stability of computational PPI prediction models still need improvement, especially for species with limited verified PPI data. Protein embeddings generated by protein language models can extract features from protein sequences and reflect hierarchical biological structures, making them suitable for predicting PPIs. Therefore, in this study, we propose a novel protein sequence-based PPI prediction framework designed for generalized PPI assessment by integrating two protein language models (PLMs) and an enhanced deep neural network (MPIDNN-GPPI). Specifically, the sequences are embedded using two protein language models, Ankh and ESM-2. A deep neural network is then used to learn representations from the feature vectors produced by PLMs. Subsequently, a multi-head attention mechanism is introduced to capture long-range dependencies and fuse them with DNN-derived representations. Finally, a deep neural network is applied to assess the probability of interaction between two proteins. To evaluate the performance of MPIDNN-GPPI, nine PPI datasets were collected from the STRING database, covering a diverse set of species: five datasets from mammals (*D. melanogaster*, *C. elegan*s, *S. cerevisiae*, *H. sapiens,* and *M. musculus*), and four datasets from plants (*O. sativa*, *A. thaliana*, *G. max*, and *Z. mays*). When trained on *H. sapiens*, MPIDNN-GPPI achieved AUC values of 0.959, 0.966, 0.954, and 0.916 on independent test sets for *M. musculus*, *D. melanogaster*, *C. elegans*, and *S. cerevisiae*, respectively. These results represent the best performance among all PPI models compared in this study. Similarly, when trained on *O. sativa*, the model achieved AUC values of 0.96, 0.95, and 0.913 on independent datasets for *A. thaliana*, *G. max*, and *Z. mays*, respectively. Ablation experiments demonstrated that models combining Ankh and ESM-2 outperformed those relying on a single protein language model. Furthermore, MPIDNN-GPPI, which incorporates multi-head attention and deep neural networks (DNN), achieved superior performance compared to models using DNN alone. These findings indicate that MPIDNN-GPPI possesses strong generalization capability for cross-species PPI prediction. The proposed model, trained on one species, can be effectively applied to accurately predict PPIs in other species.

**Supplementary Information:**

The online version contains supplementary material available at 10.1186/s12864-025-12228-y.

## Introduction

Proteins play crucial roles in regulating a wide range of cellular processes and act as the ultimate executors of cellular functions. Most proteins perform their functions in conjunction with other proteins, rather than in isolation [1]. PPIs not only contribute to a better understanding of the biological functions of uncharacterized proteins but also provide essential information for further comprehension of their biological activities [2]. For example, plant signaling [3], the plant stress response [4], the development of plant defense systems [5], and the formation of corresponding cellular organs [6] all rely on PPIs. In animals, PPIs also play important roles in protein phosphorylation [7], cytoskeleton assembly [8] and the activation of transcriptional proteins. In short, accurate and efficient identification of PPIs contributes not only to a deeper understanding of cellular life processes but also has significant implications for the development of new varieties and the investigation of disease mechanisms [9]. Therefore, an in-depth exploration of PPIs is essential for a thorough understanding of protein functions and genetic mechanisms.

Traditional biological experimental methods, such as yeast two-hybrid [10], tandem affinity purification [11], and mass spectrometry [12], are commonly used to identify PPIs. However, these methods are often costly, time-consuming, labor-intensive, lack-stability, and unsuitable for large-scale prediction tasks [13]. In addition, the functional annotation of genes and the elucidation of molecular mechanisms are fundamental goals in genomics. Central to this endeavor is the mapping of protein–protein interaction (PPI) networks. Computational prediction methods are thus essential to bridge this gap and generate testable biological hypotheses on a genomic scale.

Over the past few decades, a significant amount of protein interaction data has been amassed using high-throughput techniques such as mass spectrometry [14] and protein microarrays [15], leading to the creation of numerous databases dedicated to protein interaction research. More than 100 online databases are currently available [16], including the Search Tool for Recurring Instances of Neighboring Genes (STRING) [17], the Biological General Repository for Interaction Datasets (BioGRID) [18], BIND [19], MINT [20], and IntAct [21]. The public availability of such highly annotated data opens new horizons for the development of computational methods for PPI analysis.

In recent years, advances in machine learning and artificial intelligence have facilitated the development of various computational methods for predicting protein–protein interactions (PPIs). These methods aim to predict previously unknown interaction pairs by integrating and analyzing known PPI data to uncover potential connections. Compared to experimental approaches, computational methods offer higher sensitivity, straightforward, and capable of rapidly predicting interactions across thousands of protein pairs. These advantages have attracted significant research interest. Historically, the 3-dimensional structure of proteins served as a fundamental feature for PPI prediction. However, the identification of intrinsically disordered proteins, whose conformations vary over different time scales [22], has led to a shift in perspective. The 3-dimensional structure of proteins is no longer regarded as the sole determinant for PPIs. Instead, the primary protein structures and amino acid sequences might offer more predictive information [23]. Although protein sequence data are abundant, experimentally validated PPIs remain relatively scarce. This imbalance poses a challenge for developing reliable machine learning models, which generally require large amounts of high-quality labeled data for accurate prediction. While protein sequences are linear chains of amino acids, their interactions involve both local and long-range dependencies, complicating the extraction of discriminative features a complex task. As a result, sequence-based methods have gained prominence [24], utilizing machine learning algorithms such as support vector machines [1, 25], random forests [26, 27], *K*-nearest neighbors [28], hidden Markov models [29], and neural networks [30] for PPI prediction. Nonetheless, many of these methods still depend on hand-crafted feature extraction and selection, which is not only labor-intensive but also requires substantial domain expertise. As a major branch of machine learning, deep learning consists of multilayer neural networks capable of automatically learning stable and high-dimensional features that facilitate the interpretation of biological data structures [31]. In recent years, deep learning algorithms have been widely used to predict PPIs. For example, Hashemifar et al*.* proposed a PPI prediction model named DPPI [32], which integrates a random projection module into a convolutional neural network (CNN), achieving an accuracy of 0.9455 on a dataset comprising 11 species. Wang et al*.* developed a deep learning-based method named DeepViral [33], to predict human-virus PPIs, which reached an AUC value of 0.813. Mahapatra et al*.* [34] developed a hybrid classifier by combining a functional-link Siamese neural network with a light gradient boosting machine, which achieved accuracy values of 0.9870 and 0.9838 on intraspecies PPI datasets of *Saccharomyces cerevisiae* and *Helicobacter pylori*, respectively. Hu et al*.* presented a deep learning-based model utilizing multiple parallel convolutional neural networks, DeepTrio [35], to predict PPIs from raw protein sequences, which reached an accuracy of 0.9755 and an MCC value of 0.9515 on a yeast dataset. However, establishing these deep learning-based PPI prediction models requires coding methods to encode the amino acid sequence into digital information. In addition, existing PPI information may result in limited training data that are not representative enough to ensure robust, generalizable, and stable model performance. Pretrained protein language models (PLMs), which encapsulate a significant amount of biological prior knowledge, can help alleviate this problem. Currently, protein language models are typically trained to predict the spatial structure of proteins, enabling them to efficiently learn the representation space from protein sequences and reflect the multilevel biological structure [36]. Several studies have demonstrated the utility of PLM-derived features for PPI prediction. For example, Dong et al. developed a multitask transfer learning approach [37] using the UniRep model [38] to learn protein representations, achieving competitive results on 13 benchmark datasets. Sledzieski et al*.* proposed a PPI prediction model named D-SCRIPT, combining a deep language model with bidirectional long short-term memory, which achieved high accuracy with limited training data [39]. In our previous study, we developed a PPI prediction model using the evolutionary-scaled language modeling-2 (ESM-2) with a deep neural network, which achieved high predictive and generalized performance [40]. The existing PPI prediction models established with protein language models can learn multi-level biological features from protein sequences and improve prediction performance. However, accuracy and generalizability, especially for data-scarce species, require further enhancement.

To develop a high-performance and generalizable PPI prediction method, we introduce three key innovations in our proposed framework. First, the pretrained protein language model can capture the dependence between amino acids of a protein sequence. The PLM of Ankh is the initial general-purpose PLM trained on Google's TPU-v4, surpassing the state-of-the-art performance with fewer parameters [41]. ESM-2 is another state-of-the-art protein language model that achieves better performance in describing protein sequences [42]. As these two protein language models operate on different principles, their learned feature representations are complementary. Therefore, we can fuse the PLMs of Ankh and Esm2 to embed protein sequences to learn more essential patterns of protein sequences. Second, given the protein sequence’s long-range correlation, the multi-head attention mechanism is better suited to capturing internal feature relevance, especially long-range dependencies [43]. As a result, we introduce a multi-head attention mechanism to extract long-range dependencies within protein sequences. Third, for a deep neural network, as the depth of a deep neural network increases, the risk of overfitting increases, and the attention to key features may decrease. The self-attention mechanism can adjust feature weights by dynamically focusing on the key residual connections within the sequence to avoid falling into a local optimum. Thus, we integrate a self-attention mechanism into DNN to enhance feature representation and improve the estimation of interaction probability between protein pairs.

**Fig. 1:**
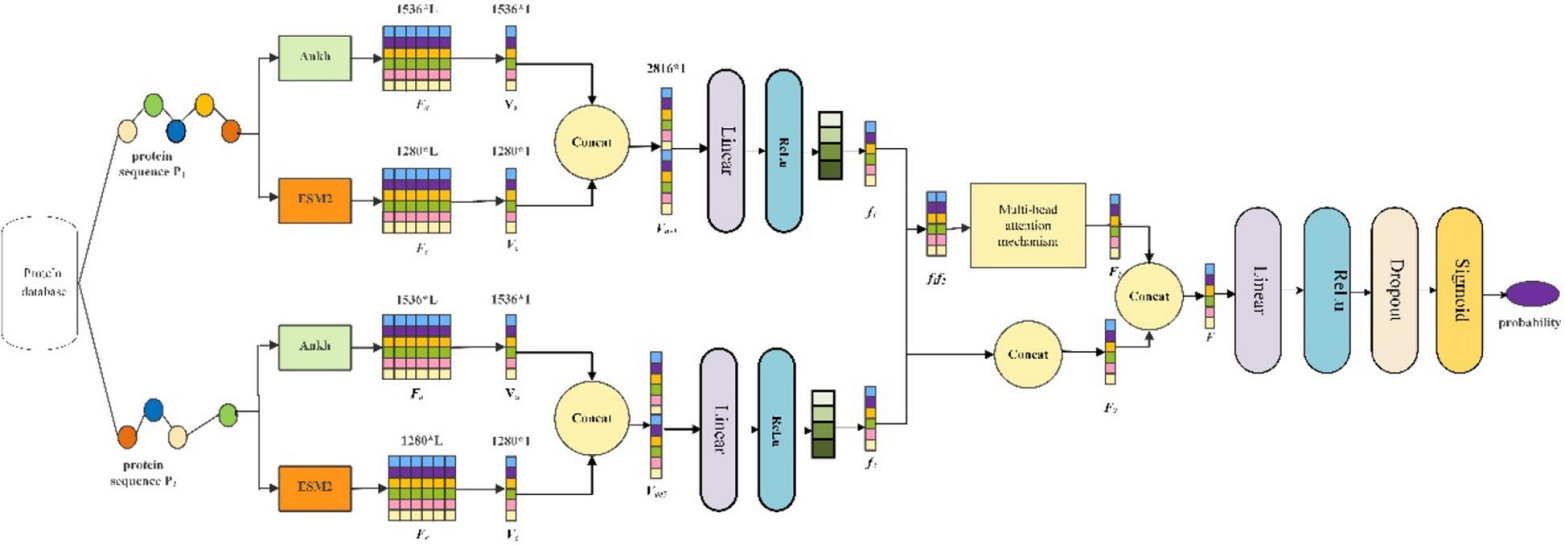
Flow chart of the generalized PPI prediction network, MPIDNN-GPPI

For these reasons, we propose a novel framework called MPIDNN-GPPI for generalized protein‒protein interaction prediction. It entails combining the recent protein language models of Ankh [41] and Esm2 [42] for protein representation and integrating a multi-head attention mechanism with a deep neural network for PPI prediction. Specifically, we first use the protein language models, Ankh and Esm2, to generate embeddings for protein sequences. The interaction information between protein pairs was then learned through a deep neural network. Next, a multi-head attention mechanism is utilized to capture long-range dependencies within the sequences. Finally, the features extracted by the DNN and the multi-head attention mechanism are fused and served as input to an enhanced DNN architecture, which incorporates a self-attention mechanism to predict PPIs. To evaluate the generalization capability of MPIDNN-GPPI, its performance was assessed on nine PPI datasets. The *H. sapiens* dataset is used as the training set, and the other four independent datasets of *M. musculus*, *D. melanogaster*, *C. elegans,* and *S. cerevisiae* are applied as the testing sets. Similarly, the *O. sativa* dataset was used as the training set, and the other three independent datasets, *A. thaliana*, *G. max*, and *Z. mays*, were used as the testing sets. The results demonstrate that MPIDNN-GPPI has better generalizability for PPI prediction compared to the other eight PPI prediction models established in this study. The overall workflow of the proposed PPI prediction method is shown in Fig. 1.

The main contributions of this study are summarized as follows:

1. We developed a high-performance and generalized PPI prediction framework using protein sequences. This framework is a fully automatic, robust, highly generalization. It combined with a multi-head attention mechanism which can accurately predict PPIs on independent datasets.
2. The proposed model combines two pretrained PLMs, Ankh and ESM-2, with a multi-head attention mechanism for PPI prediction. This integration enhances the model’s accuracy and generalizability when applied to independent datasets from both animal and plant species.
3. The model achieves high performance across different species without requiring retraining, which significantly reduces the computational time, and is therefore particularly suitable for species with limited available PPI data.

This paper is organized as follows. The first section is the "Introduction", which summarizes the motivation for developing a high-performance and generalizable PPI prediction model, reviews the current outline and recent advancements in the PPI prediction field, introduces the method developed in this paper, and outlines the main contributions of this paper and the organization of this paper. The second section presents the "Materials and Methods", which describes the dataset information used in this paper and the method we propose. The third section presents the “Results”, which describes the experimental findings, including a comparative analysis of the model constructed using our proposed method against other methods on various datasets, as well as the performance of ablation experiment models. The fourth section, "Discussion", discusses the content of this paper in detail and summarizes the limitations of the proposed method, and suggests potential directions for future research. The fifth section, "Conclusion", summarizes the content of this article.

## Materials and methods

### Materials

This study utilized nine protein–protein interaction datasets, comprising three from lower organisms (*D. melanogaster*, *C. elegans*, and *S. cerevisiae*), two from mammals *(H. sapiens* and *M. musculus*), and four from plants (*O. sativa*, *A. thaliana*, *G. max*, and *Z. mays*). All data were retrieved from the publicly available database of STRING [17]. For each dataset, protein pairs are associated with a confidence coefficient, which is calculated through the STRING assessment system. These interaction scores are normalized to a range between 0 and 1. Protein pairs with an interaction score below 0.4 were considered non-interaction pairs. Pairs with an interaction score above 0.8 were labeled as interaction pairs.

To select protein pairs with high-confidence physical protein interactions, we restrict the dataset to those interactions supported by experimental evidence scores, which indicate evidence derived from laboratory experiments. Protein pairs were excluded if the amino acid sequence length of either protein was less than 50 or greater than 800, the protein pairs were excluded. Proteins shorter than 50 amino acids typically lack the structural complexity necessary for reliable interaction prediction. To maintain computational efficiency and feasibility, proteins exceeding 800 amino acids were omitted due to GPU memory constraints. Subsequently, the CD-HIT method [44] was applied to cluster proteins at a 40% sequence similarity threshold. Furthermore, to account for the notion that genuine positive PPIs may be scarce, the ratio of interaction to non-interaction pairs was set to 1:10 [50], resulting in the compilation of nine datasets. The number of protein pairs in each of the nine datasets is shown in Table 1.

**Table 1:** Numbers of protein pairs in nine datasets that were collected and used in this study

Datasets	Interaction pairs	Noninteraction pairs	Total
*H. Sapiens*	47,932	479,320	527,252
*M. musculus*	5000	50,000	55,000
*D. melanogaster*	5000	50,000	55,000
*C. elegans*	5000	50,000	55,000
*S. cerevisiae*	5000	50,000	55,000
*O. sativa*	20,000	200,000	220,000
*A. thaliana*	5000	50,000	55,000
*G. max*	5000	50,000	55,000
*Z. mays*	5000	50,000	55,000

### Methods

**Fig. 2:**
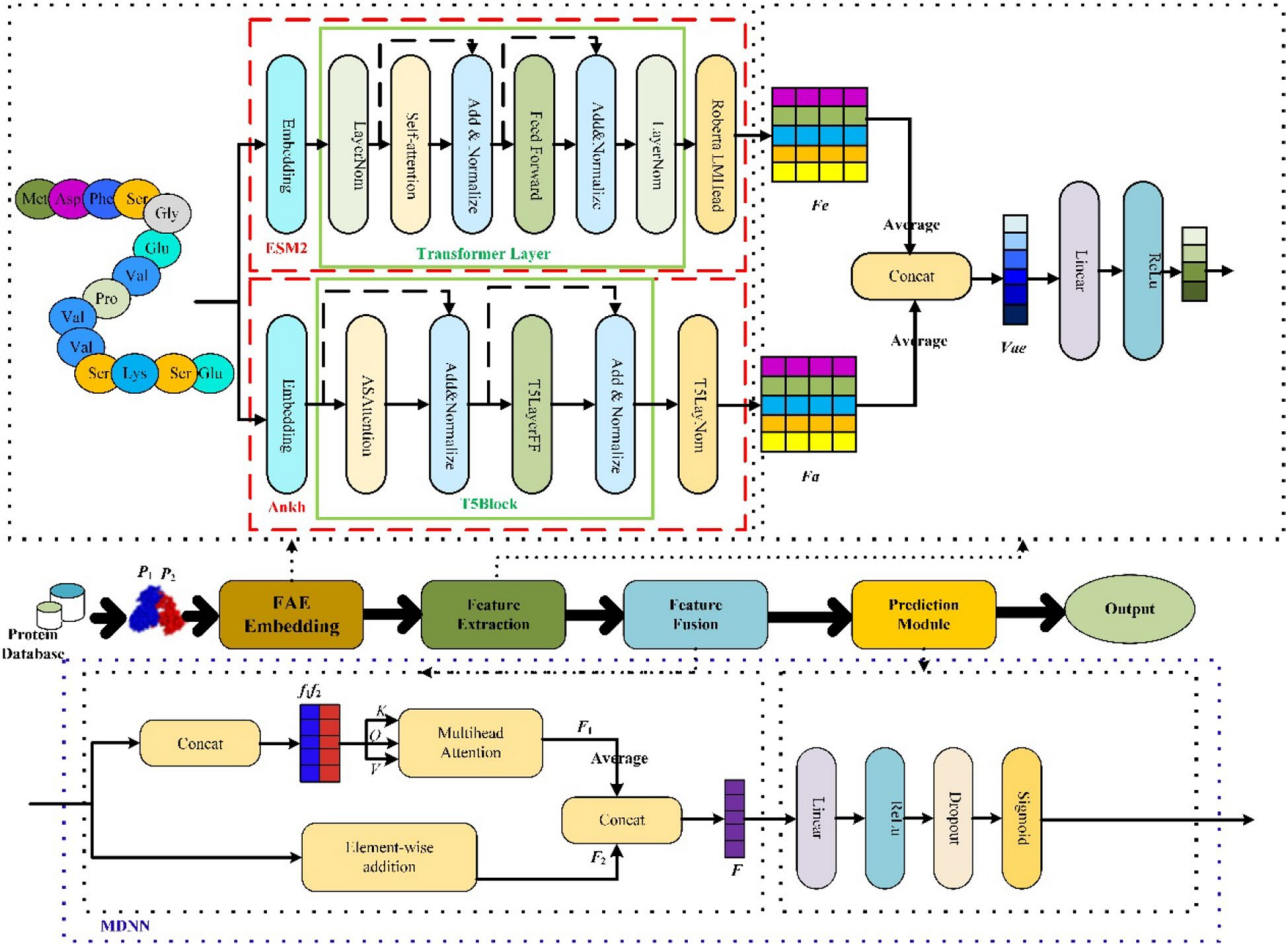
Structure of the generalized PPI prediction network, MPIDNN-GPPI

In this section, we describe the detailed architecture of the proposed model in this study. The architecture comprises four main components: the MLP embedding module, feature extraction module, feature fusion module and prediction module, as shown in Fig. 2. In our study, we harnessed the robust representational capabilities of the pretrained Ankh and ESM2 models, both of which excel at capturing contextual information and amino acid dependencies in protein sequences. We can extract latent interaction information from protein sequences by integrating these models with the Multi-Head Attention mechanism. This approach enables us to identify not only the unique characteristics of each amino acid but also accurately model both global and local sequence connections, thereby enhancing the accuracy of PPI prediction.

#### Feature embedding module

In this module, protein sequence pairs of (P_1_, P_2_) are input into protein language models of Ankh and Esm2, which convert the original protein sequences into protein representations. Specifically, each of two protein sequences is input separately into the ESM-2 model. The input protein sequence is embedded in words to generate the context perception of the sequence. Then, the embedded sequence is deeply encoded with a series of transformer layers. Next, the feature information of the protein sequence is mapped into a vector space to preserve the structural information and obtain the coding information of the protein sequence, as formalized in Eq. (1). As a result, a 1280* L-dimensional feature matrix, *F*_*e*_, was computed for each protein sequence, where *L* denotes the length of the protein sequence.

1 $${F}_{e}=RobertaLMHead\left(Transformer\left(Embedding\left(P\right)\right)\right)$$

On the other hand, each of the two protein sequences is input into the pretrained protein language model of Ankh. Similarly, the input protein sequence is embedded in words and encoded with the T5Block layer. Then, the feature information of the protein sequence is mapped into the vector space, as formalized in Eq. (2). Consequently, a 1536* L-dimensional feature matrix,* F*_*a*_, was computed for each protein sequence with the PLM of Ankh, where* L* is the length of the protein sequence.

2 $${F}_{a}=T5 Layer Norm\left(T5Block\left(Embedding\left(P\right)\right)\right)$$

Subsequently, each row of the matrices *F*_*a*_ and *F*_*e*_ is averaged to compute feature vectors. Thus, for each protein sequence, two feature vectors *V*_*a*_ and *V*_*e*_, with dimensions of 1280*1 and 1536*1, are computed from *F*_*a*_ and *F*_*e,*_ respectively.

Next, *V*_*a*_ and *V*_*e*_ are concatenated into a single vector denoted as *Vae*_1_, as expressed in Eq. (3). This resulted in a feature vector of dimension 2816*1 for each sequence with the ESM-2 and Ankh models. For each protein sequence pair (P1, P2), two feature vectors, *Vae*_1_ and *Vae*_2_, each with a dimension of 2816*1, are computed.

3 $${V}_{ae1}=cat\left(Average\left({F}_{a}\right),Average\left({F}_{e}\right)\right)$$

#### Feature extraction module

Further feature extraction is performed on each feature vector of *Vae*_1_ and *Vae*_2_ to capture hidden protein information of the proteins using a fully connected layer with shared weights. A deep neural network (DNN), composed of multiple interconnected computational neurons, is employed to learn high-level abstractions from the input data. The DNN receives input through its input layer, learns feature representations, nonlinearly transforms them through middle-hidden layers, and then produces predictions through the output layer [31, 45]. The activation function of ReLU [46] is used in this network, which thresholds negative values at 0 while retaining positive values. To avoid gradient disappearance and overfitting, a dropout layer is incorporated after the fully connected layer, as specified in Eq. (4).

4 $$f=\text{Dropout}\left(\text{ReLu}\left(\text{Fc}\left(P\right)\right)\right)$$

where *P* denotes the feature vector of the protein and* f* denotes the output of the fully connected layer.

After feature extraction, the features of *f*_1_ and *f*_2_ for two protein sequence representations were computed.

#### Feature fusion module

The feature fusion layer concatenates the protein features *f*_1_ and *f*_2,_ which are computed with the feature extraction module, into a joint representation.

On one hand, we concatenate the protein features* f*_1_ and *f*_2_ into a matrix (*f*_1_, *f*_2_) and compute the hidden information of this matrix via the self-attention mechanism, as shown in Eq. (5). This process yields a feature vector denoted *F*_1_ for the protein sequence pair.

5 $${F}_{1}=Attention\left(cat\left({f}_{1},{f}_{2}\right)\right)$$

The self-attention mechanism, first introduced by Google in 2017 [47], effectively captures internal feature relationships without heavy reliance on external information [43]. In this mechanism, attention weights (***W***) are computed by calculating the similarity of the Query (***Q***) and Key (***K***) after linear transformation. Next, these weights are normalized by the Soft-Max function. and finally, attention is computed from the weights and ***V***. The output of the self-attention module is the weighted sum of the feature vectors on all amino acids, as formalized in Eq. (6).

6 $$\text{Attention}\left({\varvec{Q}},{\varvec{K}},{\varvec{V}}\right)=\text{soft max}\left(\frac{{\varvec{Q}}{{\varvec{K}}}^{{\varvec{T}}}}{\sqrt{{\text{d}}_{\text{k}}}}\right){\varvec{V}}$$

On the other hand, an element-wise summation of *f*_1_ and *f*_2_ is performed, followed by averaging, to produce another feature vector *F*_2_, as described in Eq. (7). Finally, the two fused feature vectors *F*_1_ and* F*_2_ are concatenated to form the overall fused representation, *F*, given by Eq. (8).

7 $${F}_{2}=\frac{Element Wise Addition\left({f}_{1},{f}_{2}\right)}{2}$$

8 $$F=\text{cat}\left({F}_{1},{F}_{2}\right)$$

where *f*_1_ and *f*_2_ denote the feature vectors of the two protein sequences. *F*_1_ and *F*_2_ represent the outputs obtained through self-attention and element-wise summation with averaging, respectively, and *F* represents the final fused feature vector.

#### Prediction module

The fused feature vector *F* of the two protein sequences is input into a DNN for interaction prediction. In the prediction module, a dropout layer is used to each layer of DNN to mitigate overfitting. The final layer consists of a single neuron with a sigmoid activation function, which converts the previous layer’s output into an interaction probability score. While deep networks are capable of synthesizing diverse features, increasing the number of layers also raises the risk of overfitting and can dilute the model’s focus on salient information. To address this, a self-attention mechanism is incorporated, which dynamically emphasizes informative residual connections within the sequence. This facilitates more stable training and helps prevent the model from converging to suboptimal local minima due to overfitting.

### Evaluation metrics

To evaluate the feasibility and robustness of the PPI prediction model, five metrics, including sensitivity (Sen), precision (Pre), the Matthews correlation coefficient (MCC), the area under the precision-recall curve (AUPR), and the area under the Receiver Operating Characteristic (ROC) curve (AUC), are calculated in this study. The definitions of Sen, Pre, and MCC are provided in Eqs. (9), (10) and (11).

9 $$\text{Sensitivity}=\frac{TP}{TP+FN}$$

10 $$\text{Precision}=\frac{TP}{TP+FP}$$

11 $$\text{MCC}=\frac{TP\times TN-FP\times FN}{\sqrt{\left(TP+FP\right)\left(TP+FN\right)\left(TN+FP\right)\left(TN+FN\right)}}$$

In these formulas, *TP* (true positive) and *TN* (true negative) represent the number of correctly predicted interacting and non-interacting protein pairs, respectively. *FP* (false positive) represents the number of non-interacting protein pairs incorrectly predicted as interacting, while *FN* (false negative) indicates the number of interacting protein pairs incorrectly predicted as non-interacting.

### Experimental environment

In this study, all experiments were performed on a Linux operating system to maintain a consistent and reproducible computational environment. The detailed hyperparameter settings and model configurations are provided in Table 2 to ensure full transparency and reproducibility. To evaluate the model's performance, we employed a five-fold cross-validation method, wherein the dataset was partitioned into five subsets, each in turn served as the test set while the remaining four were used for training. This approach enhances the reliability of performance estimates and reduces the risk of overfitting.

**Table 2:** Experimental model parameters

Training Parameters	Values
Input feature size	1536
Batch size	64
Epochs	10
Optimizer	Adam
Learning rate	0.001
Loss function	BCE

To further prevent overfitting and enhance model stability, an early stopping mechanism was incorporated. Training was halted after a pre-determined number of epochs without improvement in validation performance, thereby conserving computational resources and avoiding unnecessary training while maintaining predictive accuracy.

## Results

### Performance of generalization on independent datasets

**Fig. 3:**
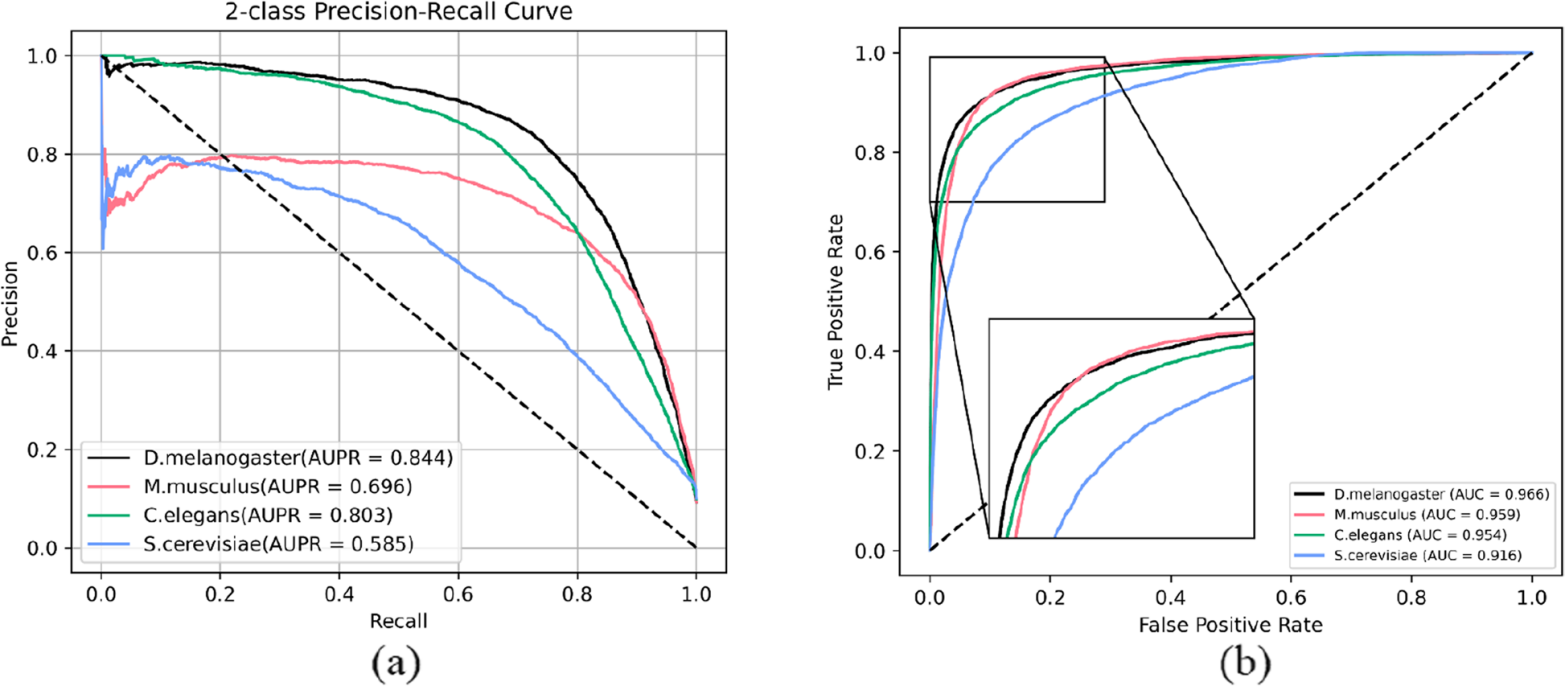
PR curves (**a**) and ROC curves (**b**) of MPIDNN-GPPI on datasets of *M. musculus*, *D. melanogaster*, *C. elegans*, and* S. cerevisiae*

To evaluate the generalization capability of MPIDNN-GPPI, the *H. sapiens* dataset was randomly divided into training and validation sets at an 8:2ratio. The *M. musculus*, *D. melanogaster*, *C. elegans*, and *S. cerevisiae* datasets were used as independent test sets. We compared MPIDNN-GPPI with three other PPI prediction models, PIPR [48], D-SCRIPT [39], and P-HYBRID [39], using the same training and test sets. The PRCs and ROCs of MPIDNN-GPPI across the four set datasets are presented in Fig. 3, and the quantitative results are listed in Table 3. In addition to the main experiments, we also conducted class-imbalance experiments to further assess the model’s performance under varying positive-to-negative ratios, as detailed in the Supplementary Information (SI). These experiments provide a more comprehensive evaluation of the robustness and generalization ability of MPIDNN-GPPI.

**Table 3:** Performance of different PPI prediction models

Species	Model	Sen (%)	Pre (%)	AUPR	AUC	F1	MCC
*M. musculus*	PIPR [48]	33.1	73.4	0.526	0.839	N/A	N/A
	D-SCRIPT [39]	34.6	81.8	0.580	0.833	N/A	N/A
	P-HYBRID [39]	35.5	82	0.609	0.838	N/A	N/A
	FAE + LR	33.9	74.1	0.572	0.875	N/A	N/A
	MPIDNN-GPPI	70.5 ± 0.0004	70.3 ± 0.00001	0.696 ± 0.0000002	0.959 ± 0.000000002	0.71 ± 0.0000006	0.68 ± 0.0000008
*D. melanogaster*	PIPR [48]	12.1	52.1	0.278	0.728	N/A	N/A
	D-SCRIPT [39]	35.9	79.8	0.552	0.824	N/A	N/A
	P-HYBRID [39]	36.1	79.8	0.562	0.824	N/A	N/A
	FAE + LR	54.3	73.7	0.685	0.926	N/A	N/A
	MPIDNN-GPPI	70.6 ± 0.0001	85.4 ± 0.00006	0.844 ± 0.00000004	0.966 ± 0.0	0.77 ± 0.0000006	0.753 ± 0.0000006
*C. elegans*	PIPR [48]	14.2	67.3	0.346	0.757	N/A	N/A
	D-SCRIPT [39]	30.6	84	0.548	0.813	N/A	N/A
	P-HYBRID [39]	30.8	84.1	0.559	0.814	N/A	N/A
	FAE + LR	46.8	80.1	0.693	0.925	N/A	N/A
	MPIDNN-GPPI	57.5 ± 0.0004	87.9 ± 0.00001	0.803 ± 0.0000002	0.954 ± 0.000000002	0.71 ± 0.0000006	0.68 ± 0.0000008
*S. cerevisiae*	PIPR [48]	8.5	39.8	0.230	0.718	N/A	N/A
	D-SCRIPT [39]	22.3	70.6	0.405	0.789	N/A	N/A
	P-HYBRID [39]	22.5	70.8	0.417	0.789	N/A	N/A
	FAE + LR	48.7	49.1	0.504	0.877	N/A	N/A
	MPIDNN-GPPI	51.7 ± 0.0001	65.5 ± 0.0002	0.585 ± 0.00000005	0.916 ± 0.000000003	0.594 ± 0.000002	0.571 ± 0.000002

The results in Table 3 demonstrate that MPIDNN-GPPI outperforms the classic PPI prediction models of PIPR, D-SCRIPT, and P-HYBRID in terms of Sen, AUPR, and AUC across all four datasets. Specifically, MPIDNN-GPPI achieved AUC values of 0.959, 0.966, 0.954, and 0.916 on the *M. musculus*, *D. melanogaster*, *C. elegans*, and *S. cerevisiae* datasets, respectively. These values represent improvements of 12% ~ 12.6%, 14.2% ~ 23.8%, 14% ~ 19.7% and 12.7% ~ 19.8% over PIPR, D-SCRIPT, and P-HYBRID across the four independent datasets, respectively. Similarly, the AUPR values of MPIDNN-GPPI are 8.7% ~ 10.7%, 28.2% ~ 56.6%, 24.4% ~ 45.7% and 16.7% ~ 35.4% higher than those of PIPR, D-SCRIPT, and P-HYBRID on the four independent datasets, respectively. Moreover, MPIDNN-GPPI showed increases in Sen of 35.1% ~ 37.5%, 34.4% ~ 58.4%, 26.4%, 43% and 29.2% ~ 43.2% compared to PIPR, D-SCRIPT, and P-HYBRID across the four independent datasets, respectively. The superior performance of MPIDNN-GPPI in both AUPR and AUC values demonstrates its capability to capture essential PPI information and its effectiveness in cross-species prediction. These results also highlight the model’s strong generalization ability and suggest that PPI prediction models can be successfully transferred across species.

### Performance of ablation experimental models on *H. sapiens* datasets

To further evaluate the reliability of MPIDNN-GPPI, we compared its performance with three existing PPI prediction models, namely PIPR, D-SCRIPT, and P-HYBRID, and a logistic regression-based model using a fivefold cross-validation method on the *H. sapiens* dataset. The results are presented in Table 4.

**Table 4:** Performance of the PPI prediction models on the *H. sapiens* dataset

Model	Sen (%)	Pre (%)	AUPR	AUC	F1	MCC
PIPR [48]	70.1	83.8	0.835	0.960	N/A	N/A
D-SCRIPT [39]	27.8	72.8	0.516	0.833	N/A	N/A
P-HYBRID [39]	40.0	*94.9*	0.844	0.962	N/A	N/A
*MPIDNN-GPPI*	***79.9*** ± 0.02	87.5 ± 0.02	***0.911*** ± 0.00002	***0.981*** ± 0.000001	**0.913** ± 0.000007	**0.827** ± 0.00003

As shown in Table 4, MPIDNN-GPPI achieves higher Sen, AUPR, and AUC values than the state-of-the-art PPI prediction models PIPR, D-SCRIPT, and P-HYBRID. In terms of Pre, MPIDNN-GPPI attains the second highest value among all ten models, which are 14.7% and 3.7% higher than D-SCRIPT and PIPR, respectively, though 7.4% lower than P-HYBRID. The AUPR of the MPIDNN-GPPI reached 0.911, exceeding those of PIPR, D-SCRIPT, and P-HYBRID by 7.6%, 39.5% and 6.7%, respectively. Furthermore, MPIDNN-GPPI achieved a Sen value of 79.9%, outperforming PIPR, D-SCRIPT, and P-HYBRID by 9.8%, 52.1% and 39.9%, respectively. MPIDNN-GPPI also attained the highest AUC among all models in this study, reaching 98.1%. These results demonstrate the effectiveness of our proposed model in predicting PPIs. The performance improvement can be primarily attributed to the incorporation of the attention mechanism, which successfully identifies and captures hidden patterns inherent in protein interactions.

**Fig. 4:**
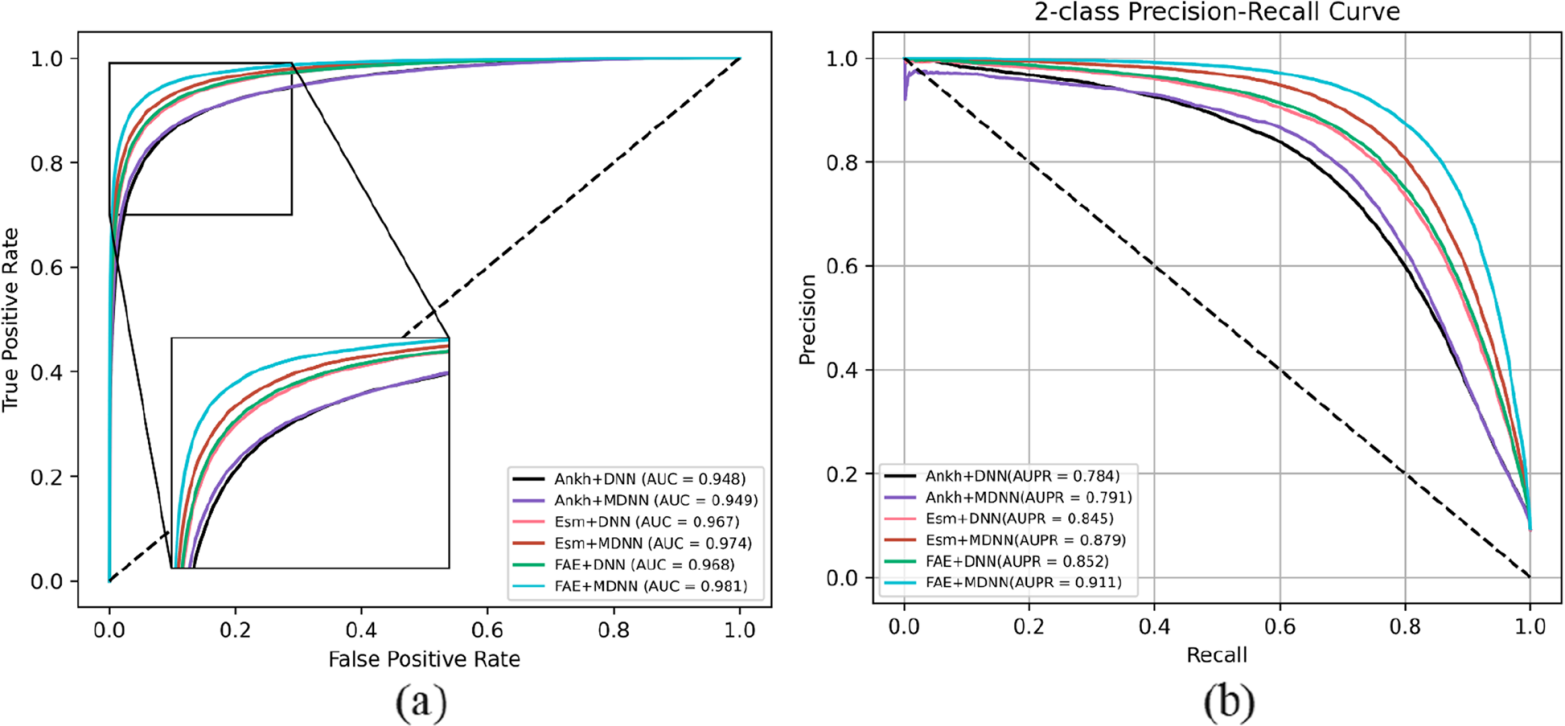
ROC (**a**) and PR curves (**b**) for ablation experiment models on the *H. sapiens* dataset

To further assess the reliability of MPIDNN-GPPI, we compared its performance against six additional PPI prediction models, namely Ankh + DNN, Esm2 + DNN, FAE + DNN, Ankh + MDNN, and Esm2 + MDNN. The results are listed in Table 5, with corresponding precision-recall curves (PRC) and ROC curves provided in Fig. 4.

**Table 5:** Ablation experiment

Model	Sen (%)	Pre (%)	AUPR	AUC	MCC
Ankh + DNN	63.9	80.9	0.784	0.948	0.695
ESM-2 + DNN	64.2	84.0	0.791	0.949	0.713
*FAE* + *DNN*	*71.8*	83.4	*0.845*	*0.967*	*0.753*
Ankh + MDNN	74.9	86.4	0.879	0.974	0.787
ESM-2 + MDNN	72.1	84.3	0.852	0.968	0.760
*MPIDNN-GPPI*	*79.9*	87.5	*0.911*	*0.981*	*0.821*

The fused feature embedding method of FAE demonstrates better performance than using either Ankh or ESM-2 alone. For instance, when FAE + DNN was used to predict PPIs, the Pre, AUPR, and AUC reached83.4%, 0.845, and 0.967, respectively, which are all higher than those achieved by Ankh + DNN or ESM-2 + DNN. Similarly, when FAE + MDNN was used to access PPIs, the values for Sen, Pre, AUPR, and AUC were 79.9%, 87.5%, 0.911, and 0.981, respectively, which are all higher than those of Ankh + MDNN or ESM-2 + MDNN. Integrating features from both Ankh and ESM-2 provides more comprehensive protein sequence information, leading to more accurate representations and improved predictive performance in PPI tasks.

Another key contribution of our framework is the integration of a multi-head attention mechanism with a deep neural network (MDNN). The MDNN-based models consistently achieve higher AUPR and AUC values than those of the DNN-based models. For example, the Ankh + MDNN model attained AUPR and AUC values of 0.879 and 0.974, respectively, compared to only 0.784 and 0.948 for the Ankh + DNN model. Similarly, the ESM-2 + MDNN model reached AUPR and AUC values of 0.852 and 0.968, outperforming the ESM-2 + DNN model, which achieved only 0.791 and 0.949. Likewise, the FAE + MDNN (namely, MPIDNN-GPPI) model achieved AUPR and AUC values of 0.911 and 0.981, exceeding the performance of the FAE + DNN model, which has AUPR and AUC values of 0.845 and 0.967, respectively. This improvement can be attributed to the multi-head attention mechanism’s ability to capture intrinsic feature relationships. By incorporating this mechanism, the network dynamically emphasizes informative residuals and adjusts feature weighting, thereby avoiding local optima and enhancing overall prediction performance.

### Generalization performance of the PPI prediction model on plant datasets

To further evaluate the generalizability and robustness of the proposed framework, we applied multiple PPI prediction models to plant datasets. The *O. sativa* dataset was divided into training and validation sets in an 8:2 ratio. Three additional plant species datasets of *A. thaliana*, *G. max,* and *Z. mays* were subsequently used as independent test sets. Five-fold cross-validation was performed on the training dataset of *O. sativa*. The performance results of the various models are presented in Table 6, and the ROC curves of MPIDNN-GPPI on the plant datasets are provided in Supplement Fig. 1.

**Table 6:** Performance of the PPI prediction models on plant-based datasets

Species	Models	Sen (%)	Pre (%)	AUPR	AUC	F1	MCC
*A* *thaliana*	FAE + LR	35.8	74.4	0.622	0.922	N/A	N/A
	Ankh + DNN	64.3	78.1	0.765	0.954	N/A	N/A
	ESM-2 + DNN	53.2	85.4	0.769	0.948	N/A	N/A
	FAE + DNN	65.8	79.1	0.791	0.955	N/A	N/A
	Ankh + MDNN	60.5	82.3	0.773	0.956	N/A	N/A
	ESM-2 + MDNN	62.6	80.3	0.776	0.949	N/A	N/A
	*MPIDNN-GPPI*	70.1 ± 0.0001	76.8 ± 0.0001	0.811 ± 0.00000001	0.960 ± 0.000000003	0.738 ± 0.000001	0.717 ± 0.000001
*G. max*	FAE + LR	28	76.2	0.586	0.910	N/A	N/A
	Ankh + DNN	53.8	77.2	0.703	0.940	N/A	N/A
	ESM-2 + DNN	47.3	84	0.720	0.933	N/A	N/A
	FAE + DNN	61.7	74.4	0.726	0.940	N/A	N/A
	Ankh + MDNN	48.7	83.5	0.727	0.945	N/A	N/A
	ESM-2 + MDNN	59.3	75.9	0.722	0.935	N/A	N/A
	*MPIDNN-GPPI*	66.1 ± 0.00005	73.63 ± 0.00008	0.771 ± 0.00000002	0.950 ± 0.000000003	0.703 ± 0.0000002	0.680 ± 0.000003
*Z. mays*	FAE + LR	16.3	73.8	0.491	0.876	N/A	N/A
	Ankh + DNN	42.8	74.2	0.611	0.909	N/A	N/A
	ESM-2 + DNN	33.1	82.6	0.605	0.887	N/A	N/A
	FAE + DNN	45.4	71.2	0.604	0.897	N/A	N/A
	Ankh + MDNN	36.1	78.4	0.614	0.910	N/A	N/A
	ESM-2 + MDNN	46.1	73.3	0.619	0.893	N/A	N/A
	*MPIDNN-GPPI*	55.7 ± 0.00009	65.8 ± 0.0002	0.658 ± 0.00000001	0.913 ± 0.000000002	0.597 ± 0.0000009	0.582 ± 0.000001
*O. sativa* (5-folds)	FAE + LR	26	74.8	0.547	0.885	N/A	N/A
	Ankh + DNN	65.6	80.5	0.770	0.948	N/A	N/A
	ESM-2 + DNN	70.4	82	0.823	0.960	N/A	N/A
	FAE + DNN	69.1	84.2	0.831	0.963	N/A	N/A
	Ankh + MDNN	65.3	86.1	0.804	0.958	N/A	N/A
	ESM-2 + MDNN	71.8	84.8	0.848	0.968	N/A	N/A
	*MPIDNN-GPPI*	72.1 ± 0.04	87.5 ± 0.05	0.879 ± 0.00006	0.977 ± 0.000004	0.896 ± 0.000006	0.794 ± 0.00003

As shown in Table 6, MPIDNN-GPPI achieves higher Sen, AUPR, and AUC across all four plant datasets compared to other PPI prediction models. Specifically, MPIDNN-GPPI attained AUC values of 0.96, 0.95, 0.913, and 0.977 for *A. thaliana*, *G. max*, *Z. mays,* and *O. sativa*, respectively. The FAE-based model consistently outperformed those based solely on Ankh- or ESM-. For instance, the FAE + DNN model showed Sen values that are 1.5%, 7.9%, 2.6% and 3.5% higher than the Ankh + DNN model, and 12.6%, 14.4%, 12.3% and 12% higher than the ESM-2 + DN model on *A. thaliana*, *G. max*, *Z. mays*, and *O. sativa*, respectively. Similarly, the AUPR of FAE + MDNN (namely, MPIDNN-GPPI) exceeded that of Ankh + MDNN by 3.8%, 4.4%, 4.4% and 7.5%, and that of ESM-2 + MDNN by 3.5%, 4.9%, 3.9% and 3.1% across the four species. respectively.

Furthermore, models incorporating the multi-head attention mechanism with a DNN(MDNN) consistently outperformed those using DNN alone. For example, on the *G. max* dataset, the AUPR values of Ankh + MDNN, ESM-2 + MDNN, and FAE + MDNN are 2.4%, 0.2% and 4.5% higher than those of Ankh + DNN, Ems + DNN, and FAE + DNN, respectively. Similarly, for *O. sativa*, the improvements are 2.7%, 2.5% and 5.2% respectively. These results demonstrate the strong generalizability of our proposed framework for predicting PPIs in plants.

**Fig. 5:**
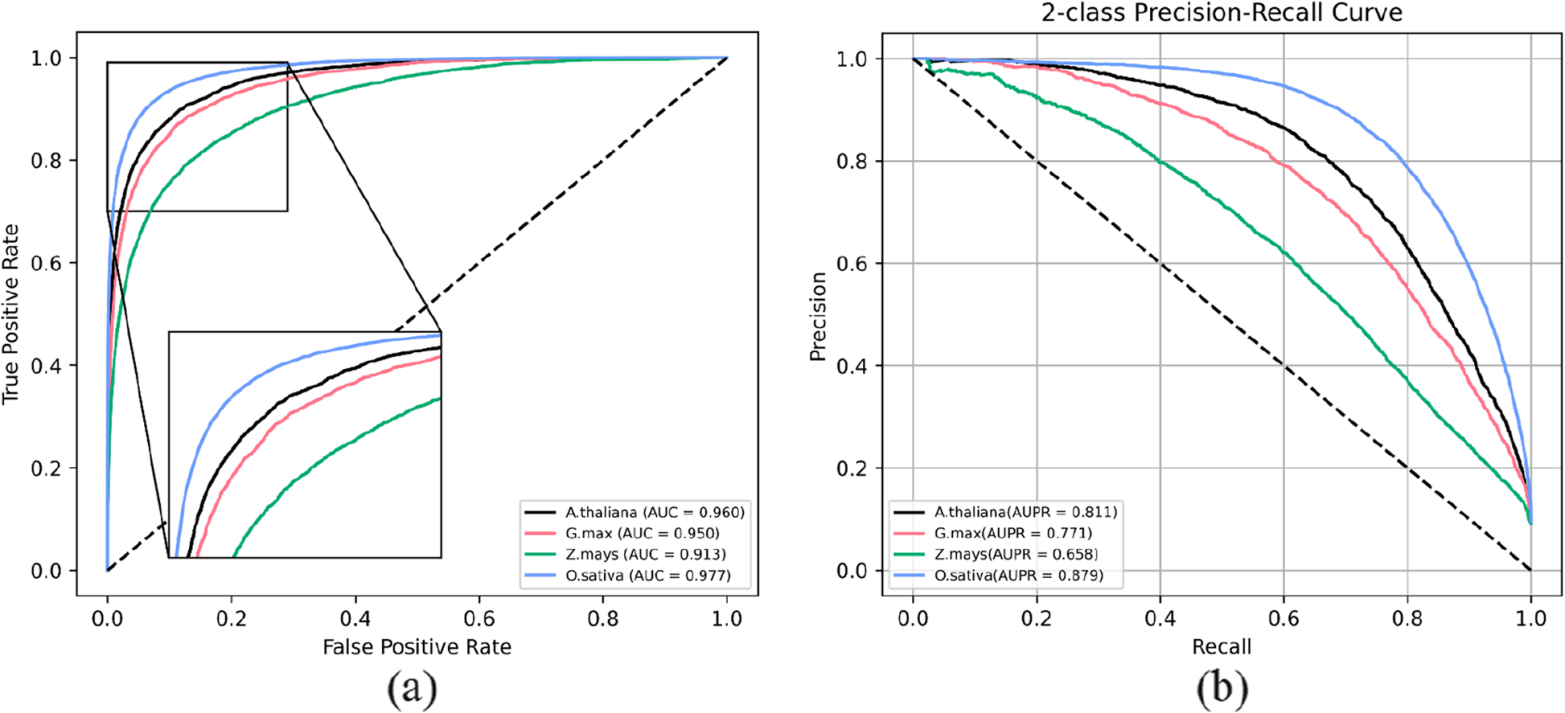
ROC (**a**) and PR curves for MPIDNN-GPPI on four plant datasets of *A. thaliana*, *G. max*, *Z. mays* and *O. sativa*

When evaluated on the *O. sativa* test set, which was derived from the same species as the training data, MPIDNN-GPPI achieved Sen, *Pre*, *AUPR*, and *AUC* values of 0.721, 0.875, 0.879, and 0.977, respectively, surpassing its performance on *A. thaliana*, *G. max,* and *Z. mays*. The ROC and PR curves for all four plant datasets are presented in Fig. 5. These findings confirm that the proposed model is highly effective for predicting plant PPIs and can facilitate the identification of potential protein interactions in plant species.

## Discussion

PPIs are of great biological importance, as they provide key clues for understanding protein functions, genetic mechanisms, and essential life activities. In recent years, a variety of sequence-based computational approaches have been developed to predict PPIs. Although state-of-the-art PPI prediction models can extract meaningful information from protein sequences, their generalization capability remains limited, particularly for species with scarce experimentally validated PPI data. In this study, we propose a novel PPI prediction framework, MPIDNN-GPPI, which demonstrates improved generalization performance on nine datasets spanning mammals, lower organisms, and plants. The main contributions of this work are as follows.

First, the proposed MPIDNN-GPPI model exhibits strong generalization across all nine datasets used in this paper. When trained and validated on *H. sapiens* data and tested on independent datasets of *M. musculus*, *D. melanogaster*, *C. elegans*, and *S. cerevisiae*, MPIDNN-GPPI outperformed PPI prediction models of PIPR [48], D-SCRIPT [39], and P-HYBRID [39] in terms of Sen, AUPR, and AUC. For the four independent datasets of *M. musculus*, *D. melanogaster*, *C. elegans* and *S. cerevisiae*, MPIDNN-GPPI achieved AUC values of0.959, 0.966, 0.954 and 0.916, which are higher than PIPR by 12%, 23.8%, 19.7% and 19.8%, higher than D-SCRIPT by 12.6%, 14.2%, 14.1% and 12.7%, and higher than P-HYBRID by 12.1%, 14.2%, 14% and 12.7%, as detailed in Table 2. Similarly, when trained on *O. sativa* and tested on *A. thaliana*, *G. max,* and *Z. mays*, MPIDNN-GPPI consistently achieved higher Sen, AUPR, and AUC values than all nine other models compared in this paper (see Table 4). These results indicate that MPIDNN-GPPI is both highly effective and widely applicable for cross-species PPI prediction, offering considerable promise for studying species with limited available PPI data.

Second, ablation studies conducted using fivefold cross-validation on the *H. sapiens and O. sativa* datasets, which revealed that the combined use of Ankh and Esm2 consistently outperformed models based on either individual protein language model. Moreover, models integrating a multi-head attention mechanism with a deep neural network surpassed those using a DNN alone. As shown in Tables 3 and 4, MPIDNN-GPPI achieved the best among all ten PPI prediction models in this paper. This improvement can be attributed to the enriched feature representation obtained by fusing Ankh and ESM-2, which captures more comprehensive and accurate PPI information than using a single model. Furthermore, the multi-head attention mechanism enhances the model’s ability to capture internal feature correlations through dynamic weight adjustment.

Third, model performance is higher when training and testing sets come from the same species compared to cross-species scenarios. For example, when evaluated on *H. sapiens* using fivefold cross-validation, the Sen, Pre, PR, and AUC values of MPIDNN-GPPI achieved 79.9%, 87.5%, 0.911, and 0.981, respectively, exceeding its performance on *M. musculus*, *D. melanogaster*, *C. elegans,* and *S. cerevisiae* datasets. Specifically, the Sen values on *H. sapiens* are 9.4%, 9.3%, 22.4% and 28.2% higher than those on *M. musculus*, *D. melanogaster*, *C. elegans* and *S. cerevisiae*, and the AUPRs on *H. sapiens* are 21.5%, 6.7%, 10.8% and 32.6% higher than those on *M. musculus*, *D. melanogaster*, *C. elegans* and *S. cerevisiae*, respectively. Similarly, when trained and tested on the *O. sativa* dataset with a fivefold cross-validation method, the MPIDNN-GPPI’s *Sen*, *Pre*, AUPR, and AUC values were 72.1%, 87.5%, 0.879 and 0.977, respectively, all of which were higher than those achieved on the *A. thaliana*, *G. max,* and *Z. mays* testing sets, as shown in Table 4. For the *O. sativa* dataset, the Sen values exceeded those for *A. thaliana*, *G. max,* and *Z. mays* by 2%, 6% and 16.4% respectively, and the AUPR values for *O. sativa* surpassed those for *A. thaliana*, *G. max,* and *Z. mays* by 6.8%, 10.8% and 22.1% respectively. One possible explanation is that proteins from the same species share more consistent interaction patterns, whereas inter-species variations introduce additional complexity and reduce prediction consistency.

In summary, we developed a generalized PPI prediction network, MPIDNN-GPPI, by integrating the protein language models Ankh and Esm2, and incorporating a multi-head attention mechanism into a deep neural network. This architecture significantly enhances prediction accuracy and generalizability across species. The model’s robustness is verified through extensive ablation experiments involving (1) individual protein models of Ankh or Esm2 and (ii) single-parameter training of the DNN. The model overcomes the challenge of cross-species prediction by fusing two pretrained protein language models. Furthermore, a multi-head attention mechanism is incorporated into the network to reduce overfitting by concentrating on key residuals and adaptively adjusting weights dynamically to avoid local optima. The proposed model achieved the highest AUC values on all nine cross-species datasets. This capability is a significant step toward large-scale functional genomics, as it allows researchers to extrapolate PPI networks from well-characterized model organisms to less-studied ones. The high performance achieved on plant species such as Z. mays and G. max is particularly promising, offering a computational strategy to accelerate research in agricultural genomics and bioengineering.

The limitations of this study are shown as follows. First, the available experimentally validated datasets remain limited in size. Second, it is necessary to develop a tool capable of reliable cross-species PPI prediction. Third, although this study used only protein sequences, other biological information, such as protein structures and multi-omics information, could provide additional relevant features. Thus, it is important to effectively integrate diverse biological data sources for improved PPI prediction in the future. Fourth, the datasets used in this paper are static, while real PPIs are dynamic and may vary across cell types and physiological states. The time series data will be further incorporated to design a dynamic interaction prediction model. Fifth, despite the PPI prediction model developed in this paper utilizing different datasets for assessment, the organisms within these datasets share co-evolutionary traits, and the PPI evolutionary pattern is conserved across different species. Thus, we will predict the PPI between different species by incorporating evolutionary models in the future. Sixth, the results presented in this study are consistently well across most datasets, demonstrating its potential applicability and robustness. While further investigation is warranted due to the observed accuracy on the *M. musculus* dataset is slightly lower, possibly due to the inherent complexity and non-linear or higher-order dependencies of some protein–protein interactions in the mouse dataset. We also recognize that the size of the training data plays a critical role in cross-species generalization. In future studies, we plan to systematically investigate cross-species PPI prediction under smaller dataset sizes and conduct a more in-depth analysis of how varying dataset sizes impact model performance, which may provide a better understanding of the model's behaviour under different data conditions. Additionally, although the current ablation experiments did not include embeddings from other large pre-trained models, we will further explore such integrations to enhance the comprehensiveness of our comparisons.

## Conclusion

This work presents a generalized PPI prediction framework capable of achieving high accuracy across diverse species, including lower organisms, mammals, and plants. By integrating two pre-trained protein language models of Ankh and ESM-2 and combining a multi-head attention mechanism with a deep neural network. The proposed MPIDNN-GPPI framework significantly enhances prediction performance and cross-species applicability. Compared to existing models, MPIDNN-GPPI provides superior accuracy and robustness. An accurate and generalizable PPI prediction framework can greatly advance our understanding of cellular life processes and disease mechanisms, ultimately facilitating the creation of new varieties and offering insights into the biological functions of uncharacterized proteins.

## Supplementary Information

Supplementary Material 1.

## Data Availability

The datasets generated and/or analyzed during the current study are available from database of STRING (https://cn.string-db.org/). The version is 11-5 (https://version-11-5.string-db.org/). In this paper, nine datasets were used, which are downloaded from this database; the specific web links are shown below.https://stringdb-downloads.org/download/protein.physical.links.v11.5/9606.protein.physical.links.v11.5.txt.gzhttps://stringdb-downloads.org/download/protein.physical.links.v11.5/10090.protein.physical.links.v11.5.txt.gzhttps://stringdb-downloads.org/download/protein.physical.links.v11.5/7227.protein.physical.links.v11.5.txt.gzhttps://stringdb-downloads.org/download/protein.physical.links.v11.5/6239.protein.physical.links.v11.5.txt.gzhttps://stringdb-downloads.org/download/protein.physical.links.v11.5/4932.protein.physical.links.v11.5.txt.gzhttps://stringdb-downloads.org/download/protein.physical.links.v11.5/4530.protein.physical.links.v11.5.txt.gzhttps://stringdb-downloads.org/download/protein.physical.links.v11.5/3847.protein.physical.links.v11.5.txt.gzhttps://stringdb-downloads.org/download/protein.physical.links.v11.5/4577.protein.physical.links.v11.5.txt.gz
