# Supplementary material for: MPIDNN-GPPI: multi-protein language model with an improved deep neural network for generalized protein‒protein interaction prediction
Source: BMC Genomics. 2025 Nov 19;26:1059. doi: 10.1186/s12864-025-12228-y (PMC12628903; doi:10.1186/s12864-025-12228-y)
Supplement: Supplementary file 1 — Supplementary Material 1. [file 12864_2025_12228_MOESM1_ESM.docx]

Supplementary Information

Supplement Table 1 Results of Five fold Training on *O.sativa* Dataset under Different

Yin Yang Ratios

| Proportion | Sen（%） | Pre（%） | MCC | AUPR | AUROC | F1 |
| --- | --- | --- | --- | --- | --- | --- |
| 1:1 | 91.8±0.2 | 96.2±0.03 | 0.883±0.0006 | 0.989±0.00001 | 0.989±0.00001 | 0.94±0.0002 |
| 1:3 | 87.1±0.1 | 96.1±0.04 | 0.889±0.0002 | 0.972±0.00001 | 0.986±0.000001 | 0.943±0.00006 |
| 1:5 | 83.1±0.08 | 89.8±0.1 | 0.838±0.0002 | 0.937±0.00008 | 0.973±0.00002 | 0.918±0.00005 |

Supplement Table 2 Results of Five fold Training on *H.sapiens* Dataset under Different Yin Yang Ratios

| Proportion | Sen（%） | Pre（%） | MCC | AUPR | AUROC | F1 |
| --- | --- | --- | --- | --- | --- | --- |
| 1:1 | 88.2±0.2 | 94.4±0.3 | 0.823±0.0005 | 0.977±0.00001 | 0.970±0.00002 | 0.912±0.0001 |
| 1:3 | 73.4±0.3 | 90.9±0.2 | 0.764±0.0006 | 0.908±0.00009 | 0.948±0.000008 | 0.872±0.0002 |
| 1:5 | 70.4±0.2 | 79.9±0.3 | 0.702±0.0001 | 0.833±0.00004 | 0.935±0.000001 | 0.849±0.00003 |

Supplement Table 3 Performance of MPIDNN-GPPI prediction model on animal datasets with different yin-yang ratios

| Species | Proportion | Sen (%) | Pre (%) | MCC | AUPR | AUC | F1 |
| --- | --- | --- | --- | --- | --- | --- | --- |
| *M. musculus* | 1:1 | 86.2 | 64.3 | 0.408 | 0.816 | 0.807 | 0.737 |
|  | 1:3 | 61.5 | 49.7 | 0.382 | 0.561 | 0.781 | 0.55 |
|  | 1:5 | 57.4±0.000009 | 56.0±0.000004 | 0.479±0.00000008 | 0.596±0.0 | 0.826±0.0 | 0.567±0.00000006 |
| *D. melanogaster* | 1:1 | 95.3 | 58.6 | 0.358 | 0.846 | 0.847 | 0.725 |
|  | 1:3 | 74.2 | 55 | 0.494 | 0.613 | 0.839 | 0.632 |
|  | 1:5 | 60.4±0.00001 | 48.5±0.00001 | 0.438±0.0000001 | 0.503±0.000000005 | 0.829±0.0 | 0.538±0.00000008 |
| *C. elegans* | 1:1 | 94.1 | 59.2 | 0.363 | 0.808 | 0.796 | 0.727 |
|  | 1:3 | 74.4 | 55.6 | 0.501 | 0.634 | 0.836 | 0.637 |
|  | 1:5 | 58.4±0.000003 | 62.8±0.00001 | 0.531±0.0000001 | 0.642±0.000000002 | 0.866±0.0 | 0.606±0.00000006 |

Continue Supplement Table 3 Performance of MPIDNN-GPPI prediction model on animal datasets with different yin-yang ratios

| Species | Proportion | Sen (%) | Pre (%) | MCC | AUPR | AUC | F1 |
| --- | --- | --- | --- | --- | --- | --- | --- |
| *S. cerevisiae* | 1:1 | 96.9 | 53.2 | 0.203 | 0.729 | 0.74 | 0.687 |
|  | 1:3 | 83.8 | 40.7 | 0.374 | 0.528 | 0.783 | 0.548 |
|  | 1:5 | 70.9±0.00002 | 38.9±0.000005 | 0.394±0.0000001 | 0.445±0.000000002 | 0.799±0.000000003 | 0.503±0.000005 |

Supplement Table 4 Performance of MPIDNN-GPPI prediction model on plant datasets with different yin-yang ratios

| Species | Proportion | Sen (%) | Pre (%) | MCC | AUPR | AUC | F1 |
| --- | --- | --- | --- | --- | --- | --- | --- |
| *A. thaliana* | 1:1 | 93.8 | 70.7 | 0.581 | 0.901 | 0.898 | 0.806 |
|  | 1:3 | 88 | 51.9 | 0.532 | 0.768 | 0.895 | 0.652 |
|  | 1:5 | 82.1±0.00002 | 51.6±0.00003 | 0.564±0.000001 | 0.741±0.0000003 | 0.915±0.0 | 0.634±0.0000006 |
| *G. max* | 1:1 | 94.6 | 69.7 | 0.573 | 0.905 | 0.904 | 0.803 |
|  | 1:3 | 89.6 | 55.4 | 0.578 | 0.773 | 0.911 | 0.685 |
|  | 1:5 | 77.7±0.00008 | 51.8±0.00004 | 0.544±0.0000005 | 0.683±0.0000003 | 0.903±0.00000001 | 0.621±0.0000003 |
| *Z. mays* | 1:1 | 97 | 62.1 | 0.456 | 0.771 | 0.807 | 0.757 |
|  | 1:3 | 86.2 | 46.8 | 0.465 | 0.698 | 0.862 | 0.607 |
|  | 1:5 | 70.8±0.00006 | 46.2±0.00006 | 0.464±0.0000007 | 0.579±0.0000001 | 0.863±0.000000002 | 0.559±0.0000004 |


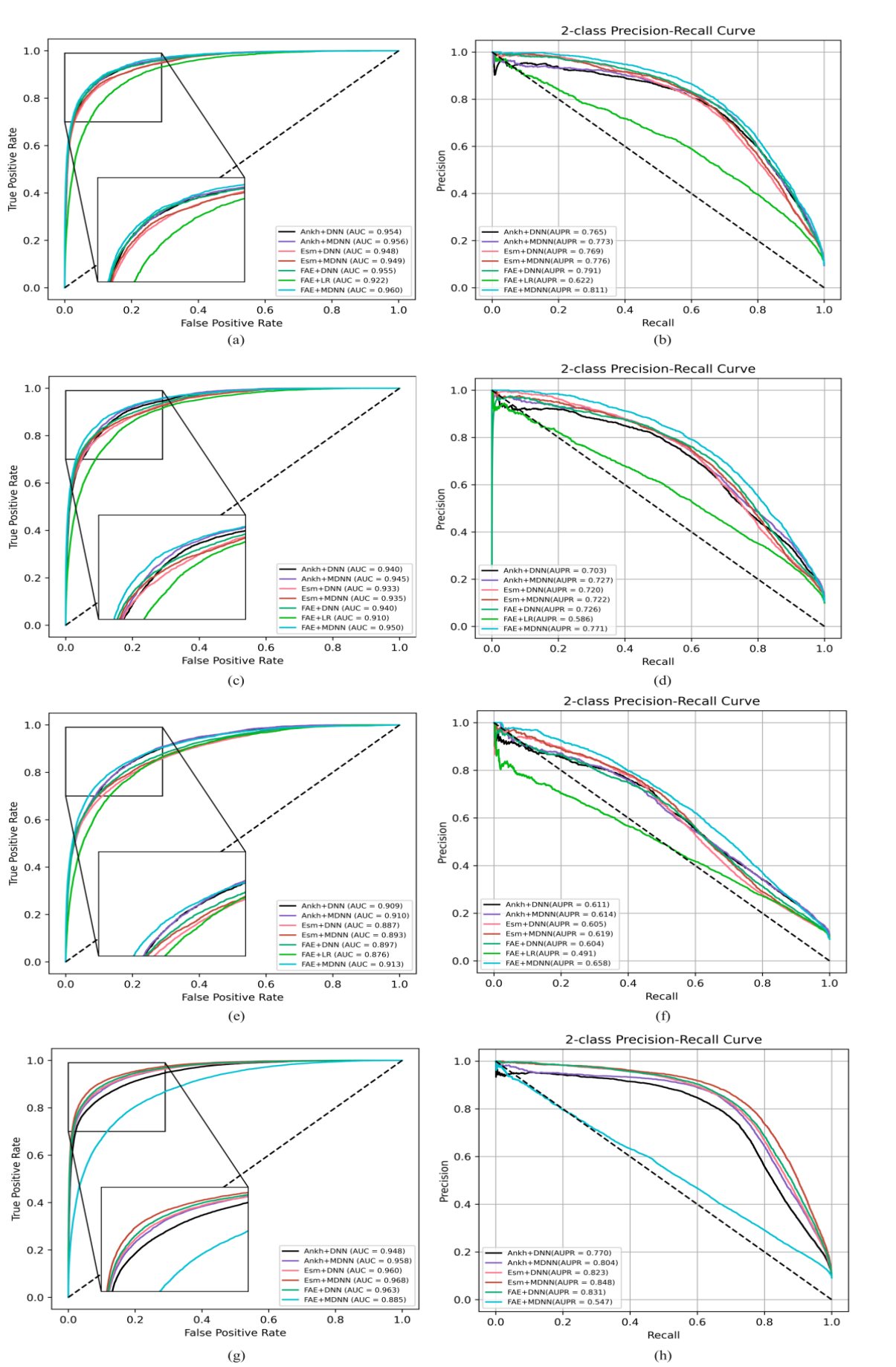


Supplement Fig. 1. ROC and PR curves for the PPI prediction models established in this study. (a), (c), (e) and (g) are the ROC curves, and (b), (d), (f) and (h) are the PR curves for the datasets of *A. thaliana*, *G. max*, *Z. mays* and *O. sativa*, respectively.
